# Organization of supragingival plaque at the micron scale

**DOI:** 10.1080/20002297.2018.1438722

**Published:** 2018-02-15

**Authors:** Ingar Olsen

**Affiliations:** ^a^ Department of Oral Biology, Faculty of Dentistry, University of Oslo, Oslo, Norway

One of the most significant advances in oral microbiology in recent years is advanced imaging methodologies to reveal the geography of oral biofilms. Although the revolution in bacterial sequencing has left us with an unprecedented abundance of data regarding the diversity and abundance of bacteria in caries and periodontal diseases, detailed information on the organization of dental biofilms in man in micrometre scale is lacking. By using spectral imaging fluorescence *in situ* hybridization guided by metagenomic sequence analysis, Mark Welch et al. [1] described a multigenus, highly organized microbial consortium in human supragingival plaque. Their unique methodology (CLASI-FISH) (see reference [2] for additional details) was developed by Gary Borisy at the Forsyth Institute, Cambridge, MA, USA. It is superior to conventional FISH which has a very limited number of different target organisms that can be detected simultaneously with standard epifluorescence or confocal laser scanning microscopy [3]. CLASI-FISH allows simultaneous differentiation of up to 28 bacterial species within a biofilm using the oral cavity as a model. It allows us for the first time to visualize the geography of biofilms, i.e. to see where specific bacteria are located and with what species they most commonly exist. This can give us insight into how bacteria interact with each other and with their hosts in health and disease.

The 10 bacterial probes applied in the current study targeted 96–98% of the cells in healthy supragingival plaque as suggested by rRNA tag sequencing data from the Human Microbiome Project (HMP) [4]. Each of the probes contained a specific oligo nucleotide conjugated to a unique fluorophore. This provided analysis of the organization of microbial communities through combinational labelling and spectral imaging. The oral, microbial biogeography (i.e. the micro-scale distribution of microorganisms that reside within the oral microbiome) thus established also allows us to understand more about the physiology and ecology of the biofilm community and its systems biology.

Admittedly, previous literature has described bacterial coadhesion or coaggregation in great detail as well as ecological succession in dental biofilm; however, we have so far lacked the micrometre-scale resolution necessary to study the spatial organization of individual bacterial cell consortia [5]. CLASI-FISH is clearly different from nucleic acid methods where the structure of plaque is destroyed. Mark Welch et al. [1] applied spectral fluorescence imaging to examine the structures produced by nine key bacterial taxa of supragingival plaque. By using sequencing data from the HMP [4], major bacterial taxa thought to be prevalent and abundant in the overall structure and function of supragingival plaque were selected and by imaging the spatial organization of these taxa, a complex, spatially organized, multigenus consortium of bacteria was detected.

**Figure 1.**
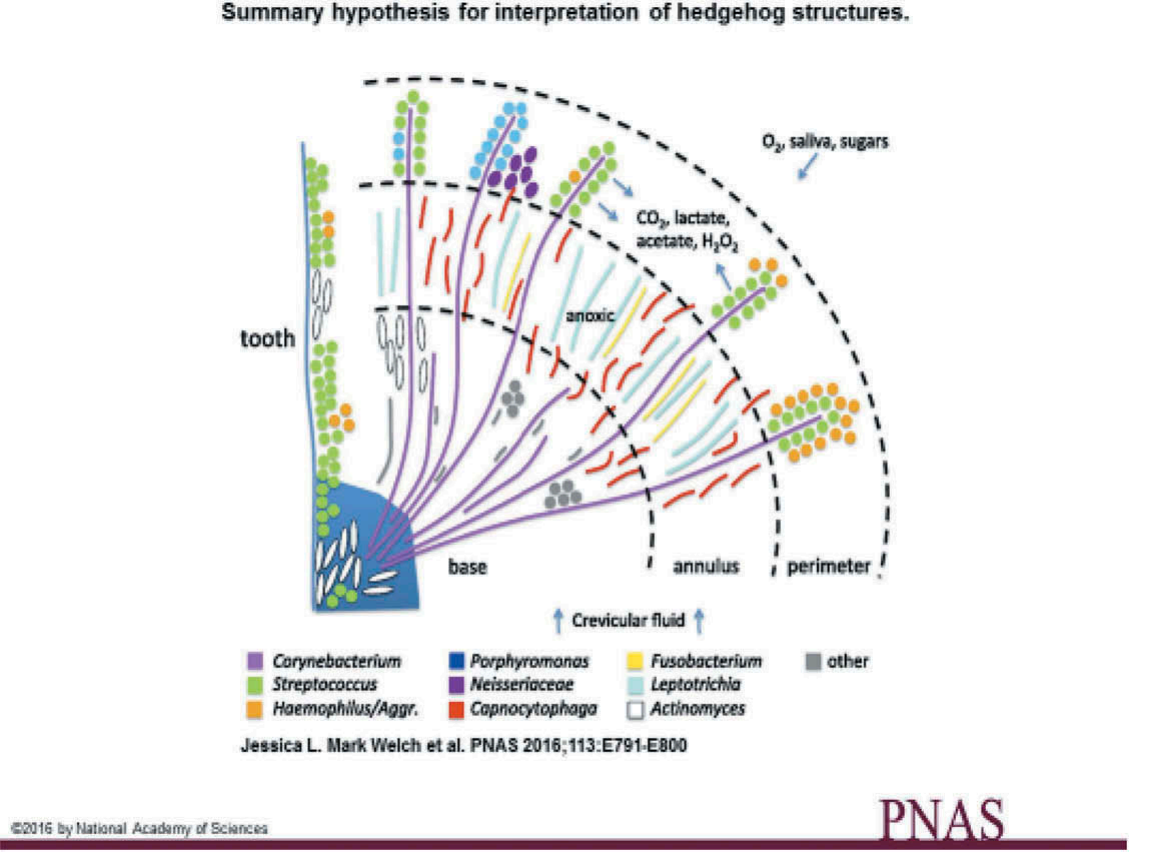
Schematic drawing of the hypothesis for interpreting hedgehog structures in supragingival plaque (from ref. [1]). Permission has been granted from PNAS.

Mark Welch et al. [1] reported that supragingival biofilm from 22 healthy volunteers consisted of a radially arranged, nine-taxon structure that was established around cells of filamentous corynebacteria. This bacterial consortium had a radius ranging from a few tens to a few hundreds of microns and was spatially differentiated. The authors described it as a hedgehog structure due to its filaments. The localization of the different taxa here suggested that they had specific functions in the consortium. As expected, anaerobic bacteria tended to localize in the interior where oxygen tension is low while facultative anaerobic and obligate aerobic bacteria tended to be at the periphery of the consortium (Figure 1). Consumers of sugars and oxygen such as streptococci produce metabolites like lactate, CO_2_, and H_2_O_2_ and tended to localize close to each other. Even if these were the most characteristic and reproducible features of the plaque structure, there was a spatial, temporal, and individual variation in the abundance of hedgehogs and other types of consortia in the supragingival plaque.

**Figure 2.**
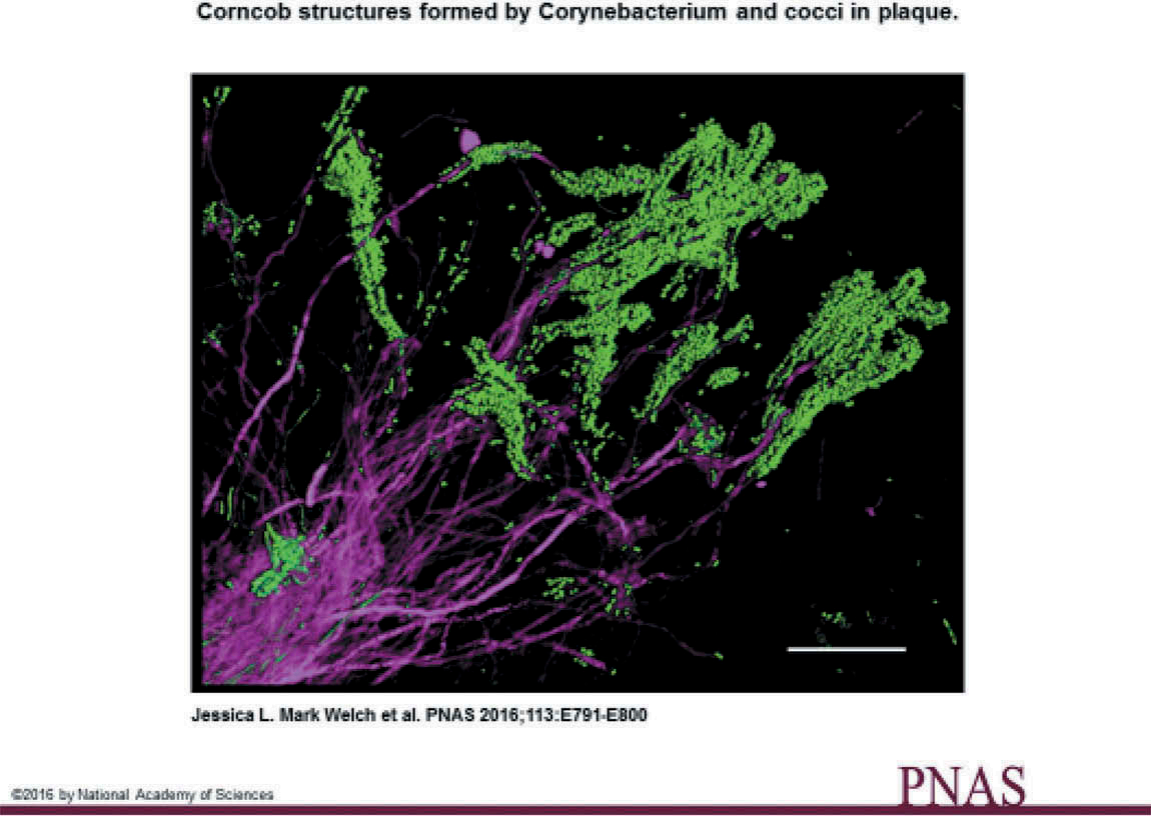
Corncob structures contain *Corynebacterium* and cocci in partially disrupted supragingival plaque. Cells of *Corynebacterium* in magenta. Cocci, bound to the tips of *Corynebacterium* filaments in green. Scale bar: 20 µm (from ref. [1]). Permission has been granted from PNAS.

The authors’ hypothesis suggested that *Corynebacterium*, which was abundant and the genus most characteristic of supragingival plaque, bound to the already existing dental biofilm substrate composed of *Streptococcus* and *Actinomyces. Corynebacterium*, projected in three planes, was found to structure the environment and create habitats for other bacteria to grow in the biofilm. Accordingly, *Corynebacterium* could be considered a bridging genus in supragingival plaque and probably more important as such than *Fusobacterium*. Corncob structures were produced at the distal tips of the *Corynebacterium* filaments (Figure 2). These structures were surrounded by coccal cells, sometimes in double layers, from *Streptococcus* and *Porphyromonas*, which were in direct contact with the *Corynebacterium* filament. Further, *Haemophilus/Aggregatibacter* established contact with *Streptococcus*, and clusters of the family *Neisseriaceae* also appeared in the periphery of the hedgehog. In the radial arrangement, intermingling taxa probably displayed a complex set of physical and metabolic interactions. Due to the *Streptococcus* cells, a micro-environment rich in CO_2_, lactate, acetate, H_2_O_2_, and low O_2_ was established. In this low O_2_/high CO_2_ environment, elongated filaments of *Fusobacteria* and *Leptotrichia* were proliferating in a so-called annulus proximal to the peripheral corncob shell. In addition, the CO_2_-loving *Capnocytophaga* was abundant in and around the filament-rich annulus. Accordingly, the hedgehog consortia were seeded into a pre-existing biofilm located on the dental surface by *Corynebacterium* filaments. Once the *Corynebacterium* filament had attached to the biofilm substrate, it seemed to play a central role organizing the consortium since it provided attachment through its surface to other bacteria in its periphery. This also implied that a complex set of physical and metabolic interactions established a framework for spatial organization of the consortium.

The relative importance of physical and metabolic influences on this arrangement remains to be established. The number of probes used was too small to obtain a full picture of the supragingival biofilm with its hundreds of species. It has been pointed out that the variability in binding efficiency and accessibility to different 16S rRNA target sites can cause false-negative binding [6]. It is unclear why the strictly anaerobic *Porphyromonas* could survive at the periphery of the hedgehog unless it was represented by relatively aerotelerant species. Certainly more refined taxonomic methods will have to be developed to determine which species of the described genera/families are present in the hedgehog structure. It will also be important to make similar studies with subgingival plaque and related diseased tissue. Studies on the microbiota of diseased tissue could increase our knowledge on what is going in microbial dysbiosis. Whether the social interactions between taxa rely on a mutualistic, commensal, or parasitic foundation will have to be elucidated.

If *C. matruchottii* is a key bacterium in the biofilm architecture of supra- and subgingival plaque, it opens up the possibility to prevent formation of mature plaque by applying antibacterial substances [7]. Furthermore, the technique may be useful for identifying key pathogens and their partners in caries and periodontitis, and thus be of value in oral microbial diagnostics. By revealing the close relationships between different bacterial cells, this technique could also be helpful in enabling culture of so far uncultivated plaque species (35%).

Noteworthy, the principles revealed on the micron-scale biogeography of supragingival plaque have relevance to the organization of microbial consortia elsewhere in the body, e.g. in the small and large intestine and in the caecum. FISH and sequencing have also been used to demonstrate that bacterial biofilms are associated with colorectal cancers and that their structural organization of the associated microbial community has a potential to contribute to progression of this disease [8].
